# Uncovering novel bacterial and archaeal diversity: genomic insights from metagenome-assembled genomes in Cuatro Cienegas, Coahuila

**DOI:** 10.3389/fmicb.2024.1369263

**Published:** 2024-05-30

**Authors:** Ulises E. Rodríguez-Cruz, Hugo G. Castelán-Sánchez, David Madrigal-Trejo, Luis E. Eguiarte, Valeria Souza

**Affiliations:** ^1^Departamento de Ecología Evolutiva, Instituto de Ecología, Universidad Nacional Autónoma de México, Ciudad de México, Mexico; ^2^Doctorado en Ciencias Biomédicas, Universidad Nacional Autónoma de México, Ciudad de México, Mexico; ^3^Department of Pathology and Laboratory Medicine, Western University, London, ON, Canada; ^4^Department of Earth, Atmospheric and Planetary Sciences, Massachusetts Institute of Technology, Cambridge, MA, United States; ^5^Centro de Estudios del Cuaternario de Fuego, Patagonia y Antártica (CEQUA), Punta Arenas, Chile

**Keywords:** Archean Eon, extreme environments, geographic isolation, microbial diversification, microbial ecology, microbial evolution, phylogenomics

## Abstract

A comprehensive study was conducted in the Cuatro Ciénegas Basin (CCB) in Coahuila, Mexico, which is known for its diversity of microorganisms and unique physicochemical properties. The study focused on the “Archaean Domes” (AD) site in the CCB, which is characterized by an abundance of hypersaline, non-lithifying microbial mats. In AD, we analyzed the small domes and circular structures using metagenome assembly genomes (MAGs) with the aim of expanding our understanding of the prokaryotic tree of life by uncovering previously unreported lineages, as well as analyzing the diversity of bacteria and archaea in the CCB. A total of 325 MAGs were identified, including 48 Archaea and 277 Bacteria. Remarkably, 22 archaea and 104 bacteria could not be classified even at the genus level, highlighting the remarkable novel diversity of the CCB. Besides, AD site exhibited significant diversity at the phylum level, with Proteobacteria being the most abundant, followed by Desulfobacteria, Spirochaetes, Bacteroidetes, Nanoarchaeota, Halobacteriota, Cyanobacteria, Planctomycetota, Verrucomicrobiota, Actinomycetes and Chloroflexi. In Archaea, the monophyletic groups of MAGs belonged to the Archaeoglobi, Aenigmarchaeota, Candidate Nanoarchaeota, and Halobacteriota. Among Bacteria, monophyletic groups were also identified, including Spirochaetes, Proteobacteria, Planctomycetes, Actinobacteria, Verrucomicrobia, Bacteroidetes, Candidate Bipolaricaulota, Desulfobacteria, and Cyanobacteria. These monophyletic groups were possibly influenced by geographic isolation, as well as the extreme and fluctuating environmental conditions in the pond AD, such as stoichiometric imbalance of C:N:P of 122:42:1, fluctuating pH (5–9.8) and high salinity (5.28% to saturation).

## 1 Introduction

In recent years, culture-independent techniques have revolutionized our understanding of microbial diversity and evolutionary relationships within the phylogenetic tree of life. Notably, the discovery of the Candidate Phyla Radiation (CPR group) by Hug et al. (2016), and the novel archaeal phylum Lokiarchaeota by Spang et al. (2015) have had profound impacts on our knowledge of microbial taxonomy, greatly expanding the phylogenomic coverage of the tree of life. More recently, Gong et al. (2022) identified several novel bacterial phyla within the FCB superphylum, highlighting the importance of MAGs in uncovering previously unknown microbial lineages and their ecological roles.

The Cuatro Ciénegas Basin (CCB) is in central Mexico, is located in the state of Coahuila, and provides a unique setting for exploring microbial diversity, spanning a valley measuring ≈30 km by 40 km at ≈740 m above sea level and is surrounded by high mountains (>3,000 m). The CCB is a closed evaporitic basin that receives ≈150 mm of annual precipitation. This basin is also characterized by its oligotrophic conditions, as ecological analyzes have revealed that a nitrogen-phosphorus ratio of 16:1 (the Redfield ratio) is common to most life on Earth (Elser, 2006). In the CCB oasis, however, these ratios are skewed due to the low level of phosphorus in the ecosystem. For example, there is a very high ratio of nitrogen to phosphorus (167:1) in the sediment of the Churince hydrological system (Souza et al., 2018). A trace of this evolutionary history has also been reported in the extreme imbalance at the bacterial intracellular level in many lineages (the most extreme being nitrogen to phosphorus ratio of 965:1 in a strain of CCB *Bacillus cereus* group) (Valdivia-Anistro et al., 2016). Despite this extreme N:P unbalance, CCB harbors an extensive system of springs, streams, and ponds of significant scientific interest and is thought to have “the highest level of endemic biodiversity in all of North America,” at least based on macroscopic organisms (70 endemic species within 500 km2) (Stein et al., 2000; Souza et al., 2006, 2012).

On the other hand, the nitrogen-phosphorus (N:P) ratio in the CCB can vary widely, from conditions of severe phosphorus deficiency to near-normal conditions, which has a direct impact on microbial proliferation (Elser et al., 2000). This environment has triggered evolutionary responses in endemic microorganisms, such as, the reduction of the genome of *Bacillus coahuilensis,* and its production of sulfolipids instead of phospholipids as potential adaptations to low phosphorus concentrations (Alcaraz et al., 2008; Souza et al., 2008; Bonilla-Rosso et al., 2012).

Overall, despite its high bacterial diversity, the Archaea domain is underrepresented at several sites in the CCB. Previous metagenomic diversity profiles in two different microbial mats in CCB (red mat and green mat, Bonilla-Rosso et al., 2012) showed that bacteria dominated in the red mat, with a relative abundance of 98%, with *Pseudomonas* as the most abundant genera, along with some representatives of Firmicutes, and Cyanobacteria, while Archaea and Eukarya represented only 1.78 and 0.26%, respectively. Similarly, at the green mat site, a relative abundance of ~93% was found for Bacteria (without a dominant phylum), only 2.06% for Archaea, and 2.79% for Eukaryota (Bonilla-Rosso et al., 2012). Using 16S rRNA gene tags (Souza et al., 2018), 5,167 OTUs (with 97% identity) were detected in soil, sediment, and water samples at different sampling sites in the Churince system in CCB (now a defunct hydrological system). This diversity represented 60 different phyla of microorganisms, of which only three belonged to the Archaea (Souza et al., 2018).

**Figure 1:**
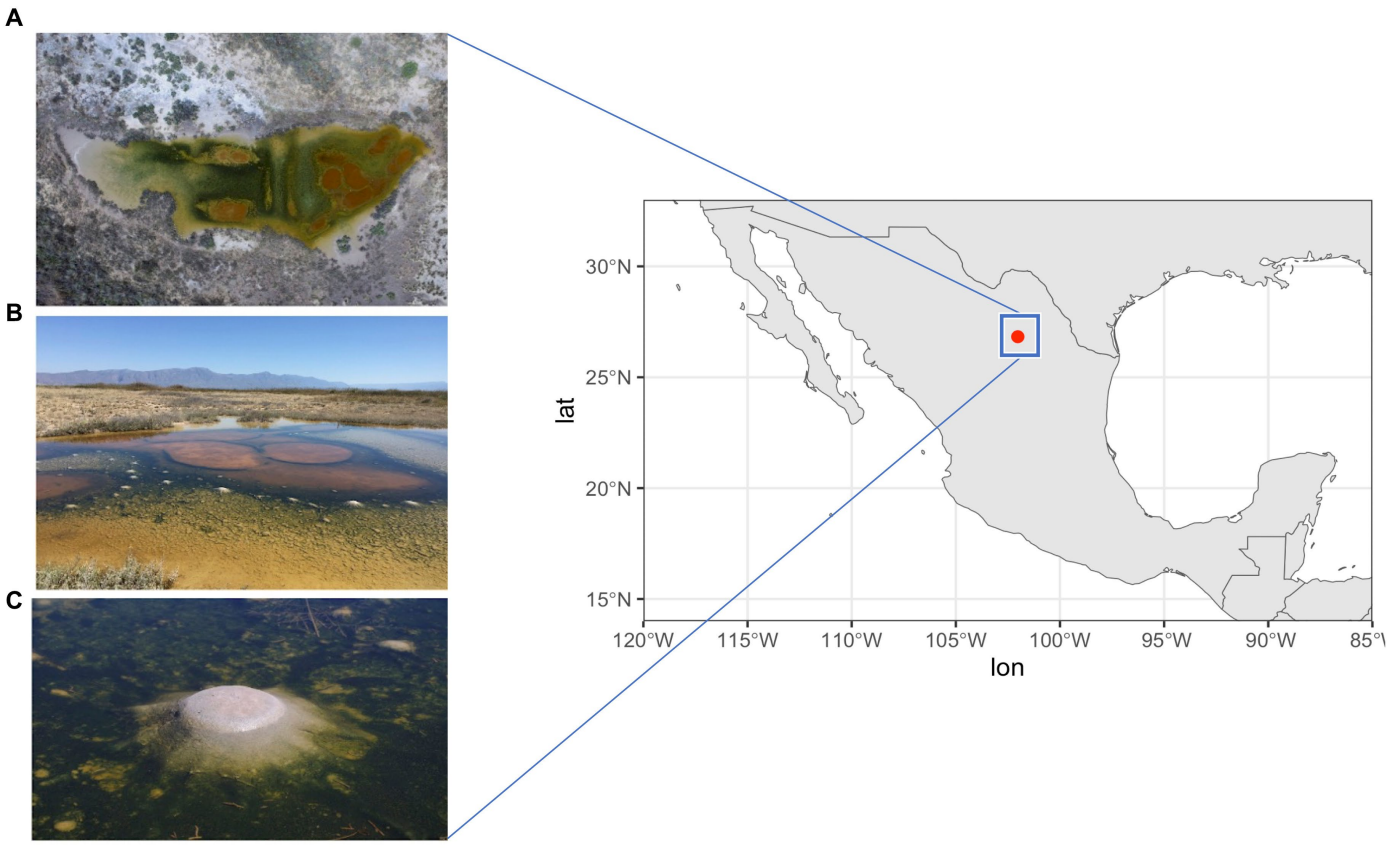
AD site in Cuatro Ciénegas Basin (CCB). **(A)** Aerial view of the site. **(B)** Photo of CCB in 2016 when the site was first explored. The orange circles mark the prominent areas of the site that were investigated. **(C)** Dome-shaped structures called Archaean Domes. Photo credit: David Jaramillo.

However, this changed in March 2016, when an unexpected rain exposed a shallow pond (see Figure 1) on the Pozas Azules ranch of Pronatura Noroeste (26° 49′ 41.7” N, 102° 01′ 28.7” W) within the CCB. This pond is characterized by dome-shaped structures that emerge around orange circles; these structures are only observed under humid conditions following a heavy rainfall. Within these elastic dome structures, there is an anoxic, carbon dioxide and methane-rich interior which reminisce what the atmosphere could have been during most of the Archean Eon, before the oxygenation of the atmospheres and oceans, hence the name “Archean domes” (AD) (see Espinosa-Asuar et al., 2022; Medina-Chávez et al., 2023; Madrigal-Trejo et al., 2023). A pH of 9.8 and a salinity of 5.28% were measured during the rainy season, while in the dry season the pH is 5 and the salinity reaches saturation. During the rainy season, a stoichiometric imbalance of C:N:P of 122:42:1 has been reported (Espinosa-Asuar et al., 2022; Medina-Chávez et al., 2023; Madrigal-Trejo et al., 2023).

An initial study conducted in 2016 on microbial mat diversity at the AD site by Medina-Chávez et al. (2023) revealed that the relative abundance of the Archaea domain reached approximately 5%, encompassing 5 Archaea phyla, 25 orders, 36 families, 93 genera and 230 species, higher than the abundances reported in analyses at other sites in the CCB mentioned above, where the relative abundance of Archaea barely reached 2.0% (Bonilla-Rosso et al., 2012; Souza et al., 2018). Subsequent studies at the AD site reported a significant increase in the relative abundance of Archaea, of ~30.60% in 2019, with the phylum Euryarchaeota being the most abundant (Madrigal-Trejo et al., 2023).

Given the high level of endemism in CCB and the remarkable diversity of Archaea in the AD site, we anticipate the discovery of numerous new lineages through the use of Metagenome-assembled genomes (MAGs). Therefore, in this study, we aim to deepen the analysis of MAGs found in the AD ponds, both in the dome formation zone and in the adjacent orange circles. Herein we show that MAGs approach provides a more comprehensive overview of the microbial diversity in AD pond than previous studies based on 16S Tags or metagenomics. Through this exhaustive analysis, we obtained a comprehensive set of 325 MAGs from the AD, 48 MAGs belonging to the Archaea domain and 277 MAGs belonging to the Bacteria domain were identified. These genomes represent a broad spectrum of previously unreported microorganisms, encompassing a total of 40 phyla, with 32 belonging to Bacteria and 8 to Archaea.

## 2 Materials and methods

### 2.1 Sample collection, genomic DNA extraction and sequencing

Sampling was conducted in the Pozas Azules ranch of Pronatura Noroeste within the CCB (26° 49′ 41.7” N, 102° 01′ 28.7” W) under SEMARNAT (Secretaria de Medio Ambiente y Recursos Naturales) scientific permit number SGPA/DGVS/03121/15. Between 2016 and 2019, fragments about 10 cm deep were collected from the surface area of domes. Starting in October 2020, our sampling approach was expanded to encompass both the domes and the orange circles at various depths, extending down to 50 cm below the surface. To ensure robust data collection, we used soil augers with 8 PVC pipes to obtain replicates for each sample.

Each sample was promptly transferred into liquid nitrogen for preservation and stored until DNA extraction was performed using the MP FastDNA™ SPIN kit for Soil following the manufacturer’s instructions. The quality of genomic DNA (gDNA) was assessed by electrophoresis on an agarose gel (1 g agarose per 100 mL buffer solution) stained with SYBR Green from ThermoFisher Scientific® using an aliquot of the sample of approximately 2 μL. Information regarding the 22 samples as well as depth, season and year of collection is provided in Table 1, it is important to note that in 2019, sampling was conducted during two different seasons (wet and dry seasons).

**Table 1:** Sample codes taken from 2016 to 2022 from the CCB AD Pond, encompassing different seasonal variations in two zones, Domes (D) and orange circles (C).

Sample	Type	Depth sampling (cm)	Sampling zone	Sampling date	Season	Raw reads	Trimmed reads	N50
D1M01	Microbial mat	0–10	Dome	Apr-16	Dry	28,859,454	26,799,269	2073
D1M02	Microbial mat	0–10	Dome	Oct-16	Wet	4,772,053	4,340,057	1821
D1M03	Microbial mat	0–10	Dome	Feb-17	Dry	8,203,484	7,269,569	1864
D1M04	Microbial mat	0–10	Dome	Oct-18	Wet	10,030,782	8,346,670	1,401
D1M05	Microbial mat	0–10	Dome	Mar-19	Dry	25,873,990	22,935,865	1726
D1M06	Microbial mat	0–10	Dome	Sep-19	Wet	20,153,088	18,787,825	1,532
C1M08	Water	0–10	Circle	Oct-20	Wet	24,065,589	19,275,870	1,667
C4M09	Sediment	30–40	Circle	Oct-20	Wet	14,315,374	13,361,648	1,152
C5M10	Sediment	40–50	Circle	Oct-20	Wet	18,050,094	16,518,645	1,138
D1M07	Microbial mat	0–10	Dome	Oct-20	Wet	17,148,993	15,124,218	1,532
D4M11	Microbial mat	30–40	Dome	Oct-20	Wet	18,976,795	17,298,109	1,354
D5M12	Microbial mat	40–50	Dome	Oct-20	Wet	16,106,607	14,999,646	1,487
D1M13	Microbial mat	0–10	Dome	Sep-21	Dry	11,669,164	7,060,584	1,480
D2M14	Microbial mat	10–20	Dome	Sep-21	Dry	6,280,722	5,893,600	1,056
D3M15	Microbial mat	20–30	Dome	Sep-21	Dry	5,849,741	3,642,579	1,065
D4M16	Microbial mat	30–40	Dome	Sep-21	Dry	4,894,276	4,657,996	755
D5M17	Microbial mat	40–50	Dome	Sep-21	Dry	2,851,518	2,706,609	884
D1M18	Microbial mat	0–10	Dome	Mar-22	Dry	1,666,524	1,593,750	907
D2M19	Microbial mat	10–20	Dome	Mar-22	Dry	1,308,223	1,264,518	1,022
D3M20	Microbial mat	20–30	Dome	Mar-22	Dry	1,055,947	1,006,567	893
D4M21	Microbial mat	30–40	Dome	Mar-22	Dry	687,386	657,723	800
D5M22	Microbial mat	40–50	Dome	Mar-22	Dry	1,152,282	1,099,173	863

Samples were sequenced at Cinvestav-Langebio^1^ Irapuato, Mexico, using Miseq Reagent Kit v3 2×300 bp paired-end on the Illumina Miseq platform.

### 2.2 Metagenomic analysis

We filtered the raw sequencing data using FastQC (v0.11.8) (Andrews, 2010) and Trimmomatic (v0.39) (Bolger et al., 2014) with the following parameters: ILLUMINACLIP:adapters.fa:2:20:10 LEADING:10 TRAILING:3 SLIDINGWINDOW:4:20 MINLEN:36. Using default parameters, the trimmed reads were then taxonomically classified using Kaiju (v1.8.1) (Menzel et al., 2016). Results were visualized using R (v4.1.0) (R Core Team, 2021) with ggplot2 (v3.3.5) (Wickham, 2016). The trimmed reads were employed to do nonpareil curves to assess the completeness of each metagenomic dataset by estimating redundancy and coverage, providing insights into the representativeness and sequencing depth required for a comprehensive microbial community analysis (Rodriguez-r and Konstantinidis, 2013).

The trimmed reads were further utilized to conduct a Pearson correlation test, examining the relationship between relative abundance at the phylum level and sampling depth, irrespective of the sampling area. This analysis was performed using R software (v4.1.0). The choice of the Pearson correlation coefficient allows for the exploration of linear relationships, providing insights into how changes in sampling depth may influence the proportional representation of different phyla.The rationale behind this analysis lies in the understanding that sampling depth can serve as a proxy for environmental gradients, reflecting diverse ecological niches within the surveyed habitats. By quantifying the correlation between phylum-level abundance and sampling depth, we seek to elucidate whether specific microbial taxa exhibit depth-dependent patterns and how these patterns may contribute to the overall microbial community structure.

Assembly of reads was performed using MetaSPAdes (v3.15.3) (Nurk et al., 2017) with the following parameters spades.py --meta −1 file_R1.fastq −2 file_R2.fastq. The assembled contigs were required for binning, which was performed using MaxBin2 (v2.2.7) (Wu et al., 2015) with default parameters: minimum contig length 1,000, max_iteration 50, and prob_threshold 0.9 for EM final classification and MetaBat2 (v2.12.1) (Kang et al., 2019) with minimum contig length (default 2,500). To reduce contamination in the bins, the software Binning refiner (v1.4.2) (Song and Thomas, 2017) was used with refined bins size larger than 512 Kbp. MAGs contamination and completeness was assessed using CheckM (v1.1.3) (Parks et al., 2015) with default settings. Quality criteria for MAGs, following MIMAG standards (Bowers et al., 2017), specify that those of good quality should have completeness greater than 70% and contamination less than 10%, while high-quality ones should achieve completeness greater than 90% and contamination less than 5%. Additionally, they should include the 23S, 16S, and 5S rRNA genes, as well as at least 18 tRNAs.

### 2.3 Phylogenetic placement of MAGs

For taxonomic assignment and placement of MAGs in the phylogenetic tree of life, we used the GTDB-tk (v2.3.2) software toolkit (Chaumeil et al., 2022), which identifies 53 and 120 archaeal and bacterial marker genes, respectively, by using HMMER (Eddy, 2011). Briefly, genomes were assigned to the domain with the highest proportion of identified marker genes. The selected domain-specific markers were aligned using HMMER, concatenated into a single multiple sequence alignment, and trimmed with the bacterial or archaeal ∼5,000 column mask used by GTDB (Chaumeil et al., 2022). This and the following phylogenetic trees were visualized with the iTOL tool (Letunic and Bork, 2021).

In addition to the previous phylogenetic taxonomic classification of MAGs, we performed a species tree encompassing all MAGs, both Archaea and Bacteria, to provide a general overview of the MAGs of the AD site. To achieve the above, the Orthofinder program (Emms and Kelly, 2019) was used, which implements the DIAMOND (Buchfink et al., 2021) program for the inference of orthologous genes, MCL (Van Dongen, 2008) for the clustering algorithm, ETE Tree library (Huerta-Cepas et al., 2016) for all tree management, MAFFT (Katoh and Standley, 2013) for multiple sequence alignment. Model selection and phylogenetic tree inference by maximum likelihood was performed with the IQ-TREE program (v2.2.2.3) using the multi-orthogroup amino acid alignment generated by orthofinder.

### 2.4 Functional annotation of the MAGs

To explore the metabolic capabilities of MAGs with respect to the C, O, N, S, and Fe biogeochemical cycles, we used the MEBS (Multigenomic Entropy Based Score) program (De Anda et al., 2017), with the parameters recommended by the developers, using the translated coding sequences for each MAG, which were obtained using the prokka software (Seemann, 2014)., In addition to the annotation in MEBS, we performed a more detailed functional annotation using freely available hidden Markov model (HMM) databases, for microbial metabolic genes of environmental/biogeochemical importance. For example, we used the metabolic-hmms database (available for free at https://github.com/banfieldlab/metabolic-hmms), FOAM (Functional Ontology Assignments for Metagenomes) (Prestat et al., 2014), TIGRFAMS (Haft, 2003) and Pfam V36.0. This annotation was mapped to KEGG orthologs and then normalized to the total number of coding sequences per genome. In R, hierarchical clustering was performed on the resulting dataset using the complete linkage method as implemented in hclust function (R Core Team, 2021).

Additional to analyze this functional annotation, we conducted a Principal Component Analysis (PCA) in R. The results of this annotation were visualized with the ggplot2 package.

### 2.5 Verification of the monophyletic clades of CCB MAGs

Given the observed monophyletic clustering patterns of some MAGs across distinct phyla, we conducted comprehensive phylogenomic analyses. The primary aim was to ascertain whether MAGs, exhibiting monophyletic clustering in the GTDB-tk reference tree, consistently displayed this pattern in our phylogenomic approach, utilizing MAGs and genomes reported in the NCBI database. This analysis is grounded in the understanding that relationships within a phylogenetic tree are shaped by shared novel characteristics (apomorphies) among analyzed taxa. These distinctive traits, acquired during the phylogenetic separation from a common ancestor, endure in the new population through genetic relationships among individuals. Monophyletic groups, defined by apomorphic character states, emerge as natural entities in the tree. Such groups denote organisms more closely related to each other than to external entities, sharing unique characteristics absent in distant ancestors (Slobodian and Pastana, 2020).

We investigated three distinct taxonomic groups (see Supplementary Tables S1–S3 for additional information about the genomes used). The first group consisted of the Candidate phylum Bipolaricaulota, characterized by its deep branching within the bacterial domain and the presence of members capable of autotrophic carbon fixation via the ancient Wood-Ljungdahl pathway for carbon fixation (Takami et al., 2012). We further explored the superphylum Planctomycetes, Verrucomicrobia, Chlamydiae (PVC), which encompasses bacteria exhibiting eukaryote-like cellular compartmentalization and varying degrees of cell organization (Kamneva et al., 2012). Lastly, we focused on Cyanobacteria, due to their pivotal role as primary producers in the microbial ecosystem, influencing carbon and nitrogen cycles (Elster and Kvíderová, 2011). Phylogenomic analyses were conducted separately for each of the three taxonomic groups. Model selection and maximum likelihood phylogenetic tree inference were performed using the IQ-TREE program (v2.2.2.3). The analysis utilized the multi-orthogroup amino acid alignment generated by OrthoFinder.

Metagenome recruitment analysis emerges as an effective tool for investigating the endemism of microbial genomes in specific environments. The low genomic similarity or sparse coverage of sequencing reads to a widely distributed reference genome suggests possible endemism. The identification of distinctive mapping patterns, combined with phylogenetic, provides evidence for inferring the endemism of a genome within the context of its natural habitat. The reference genomes were selected based on their taxonomic classification. That is, when the MAG of this study was classified at least at the genus level by the GTDB-tk software, a genome of the same taxonomic genus was selected, provided it was available in the NCBI RefSeq databases (refer to Supplementary Table S4 for information about the 17 reference genomes used in this analysis). To perform metagenomic recruitment analysis with Recruiteasy (Gerhardt et al., 2022) software, the mapping of sequencing reads to relevant reference genomes was performed using the bowtiew2 and samtools softwares. For example, the reference genome of *Halothece* sp. PCC_7,418 was compared to the D1M06 metagenome. This choice was based on MAG D1M06_8, classified as *Halothece* sp., which exhibits the highest integrity and lowest contamination (87.76 and 1.39%, respectively) among the *Halothece*-classified MAGs in the dataset, according to the parameters of the CheckM program.

## 3 Results

### 3.1 Taxonomic composition: bacteria prevail over archaea in the AD site

A total of 22 samples obtained from 2016 to 2022 were analyzed, 19 from microbial mats from the dome-shaped structures, two from sediment at different depth, and one from a water sample for the orange circle area. For a more detailed description about the samples, see Table 1.

In addition, we generated nonpareil curves for each metagenome, the results of which can be seen in Supplementary Figure S1. The x-axis shows the sequencing effort, i.e., the total amount of sequencing data produced for each sample. The y-axis measures the estimated average coverage, i.e., the proportion of the sample’s DNA that was sequenced at least once. The CCB metagenomes show variations in the estimated average coverage using nonpareil curves. Dome sample D5M22 showed an average coverage of more than 20%, like metagenomes D4M11, D4M16, D3M20, and D4M21. These data suggest that the sequencing effort for these samples may not have been sufficient to capture the full diversity of their microbial communities. Nevertheless, many metagenome-assembled genomes (MAGs) were found in these samples without a clear taxonomic assignment. This could indicate, that despite the low coverage, we obtained many novel microorganisms, suggesting that there is still much to learn about the microbial biodiversity of AD. Sadly, this site dried out in 2023 and no more samples could be recovered. On the other hand, not all dome samples had a similar estimated average coverage. Samples D1M01, D1M03, D1M07, and D3M15 had a coverage of 60%, while samples from the circles had a coverage of 80% C1M08, C4M09, C5M10; this shows us that the variability in coverage between different samples and sites could reflect significant differences in the complexity and microbial diversity of each site within Cuatro Ciénegas.

**Figure 2:**
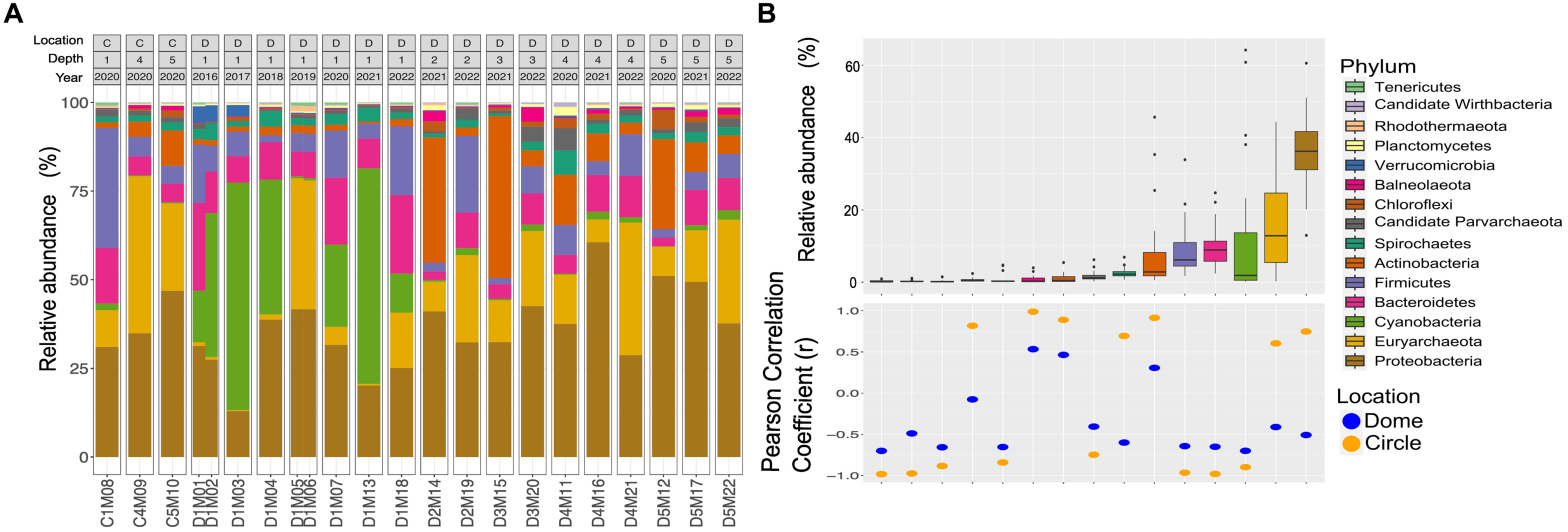
Taxonomic assignment of the sequencing reads from the AD site in CCB. **(A)** Taxonomic classification with Kaiju at the phyla level using metagenomic reads. Labels above correspond to sampling area orange circles C) or domes D), sampling depth [0–10 cm (1) to 40–50 cm (5)], and sampling year 2016 to 2022. **(B)** The relative abundance of the most representative phyla. Pearson correlation coefficient values (r) between relative abundance and sampling depth.

Regarding to the taxonomic assignment of the sequencing reads (Figures 2A,B), the 15 most abundant phyla of both Archaea and Bacteria in the 22 metagenomes were Proteobacteria (34.4%), followed by Euryarchaeota (14.45%), Firmicutes (11.31%), Bacteroidetes (11.02%), Actinobacteria (9.06%), Cyanobacteria (8.9%), Spirochaetes (2.03%), Chloroflexi (1.14%), Planctomycetes (0.99%), Candidate Parvarchaeota (0.92%), Verrucomicrobia (0.78%), Balneolaeota (0.61%), Nitrospirae (0.42%) and Tenericutes (0.38%). One of the most notable changes in the surface dome samples from 2016 to 2019 was the significant increase in the presence of Archaea, which rose from approximately 1–4% to around 33% in 2019.

Within the Archaea domain, Euryarchaeota was the only archaeal phylum with a high relative abundance in the analyzed samples. Other seven phyla (i.e., Thaumarchaeota, Nanohaloarchaeota, Candidate Lokiarchaeota, Crenarchaeota, Candidate Korarchaeota, Candidate Micrarchaeota, Nanoarchaeota) were found with relative abundance values below 1.0% for each of them.

The correlation analysis between the sampling depth and the relative abundance at the phylum level (Figure 2B) showed that some phyla are found preferentially at the surface, such as Cyanobacteria, Bacteroidetes, Firmicutes, Parvarchaea, Verrucomicrobia, Rhodothermaeota, Wirdthbacteria and Tenericutes. Likewise, other phyla, such as Balneolaeota, Chloroflexi and Actinobacteria, are found preferentially in deeper samples.

### 3.2 Metagenome-assembled genome quality

**Figure 3:**
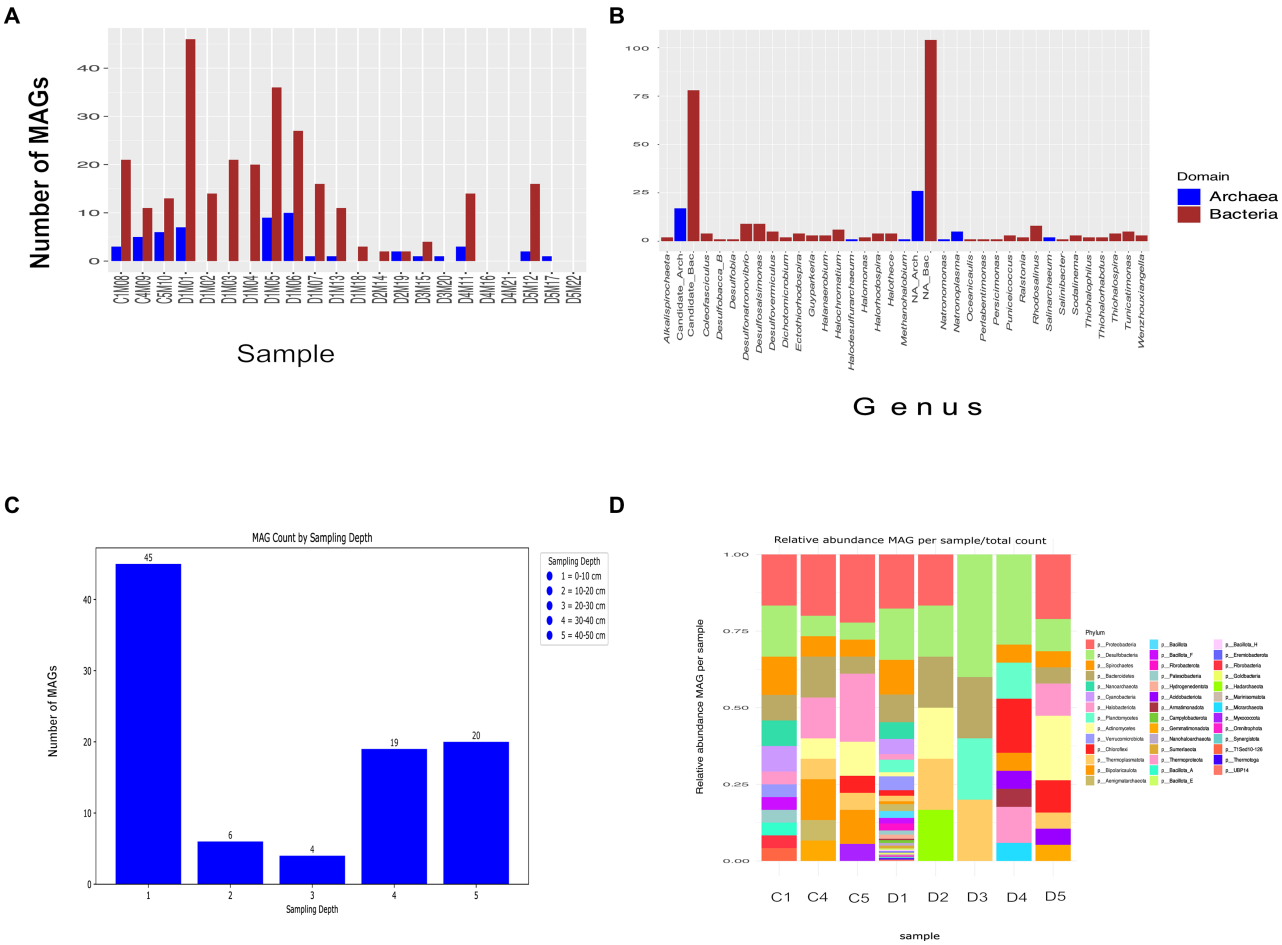
Summary of MAGs assembled from sequencing reads from the AD site in CCB. **(A)** Number of MAGs obtained per sample. **(B)** MAG classified at the genus level. **(C)** Number of MAGs obtained per sampling depth **(D)** Relative abundance MAG per sample/total count.

From the 22 metagenomes collected, an initial set of 1,044 MAGs unfiltered by completeness and contamination parameters were obtained. After filtering, 277 out of 325 MAG were classified as bacteria, and only 48 were identified as archaea. This distribution suggests a prevalence of bacteria in the microbial community of the AD site in CCB. The abundance of MAGs varied depending on the sampling depth, as illustrated in Figures 3A,C, with the highest number (245 MAGs) observed in the upper layer (0–10 cm). In contrast, only 6 MAG were detected at a depth of approximately 10 to 20 cm, 5 MAG at 20–30 cm. Compared to the previous two depths, there appears to be an increase in the number of MAGs at the depths of 30–40 cm and 40–50 cm, with 32 and 37 MAGs, respectively. For detailed information on the specific parameters associated with each MAG, readers are referred to Supplementary Table S4.

A notable diversity of microbial phyla is observed across different sampling depths. Sampling depth D1 stands out as the most diverse (Figure 3D), encompassing a total of 20 unique phyla, with Proteobacteria being the most abundant phylum and Marinisomatota the least abundant. In contrast, sampling depth D3 exhibits the lowest diversity, with only 3 unique phyla (Planctomycetes, Bacteroidestes and Thermoplasmata). Sampling depth C1 occupies an intermediate position with 12 phyla, with Spirochaetes being notably abundant and Nanoarchaeota scarcely present. Within the initial 325 MAGs, 12 met the high-quality MAGs criterion, 9 belong to Bacteria, and 3 to Archaea.

### 3.3 Phylogenetic outlook on MAGs

**Figure 4:**
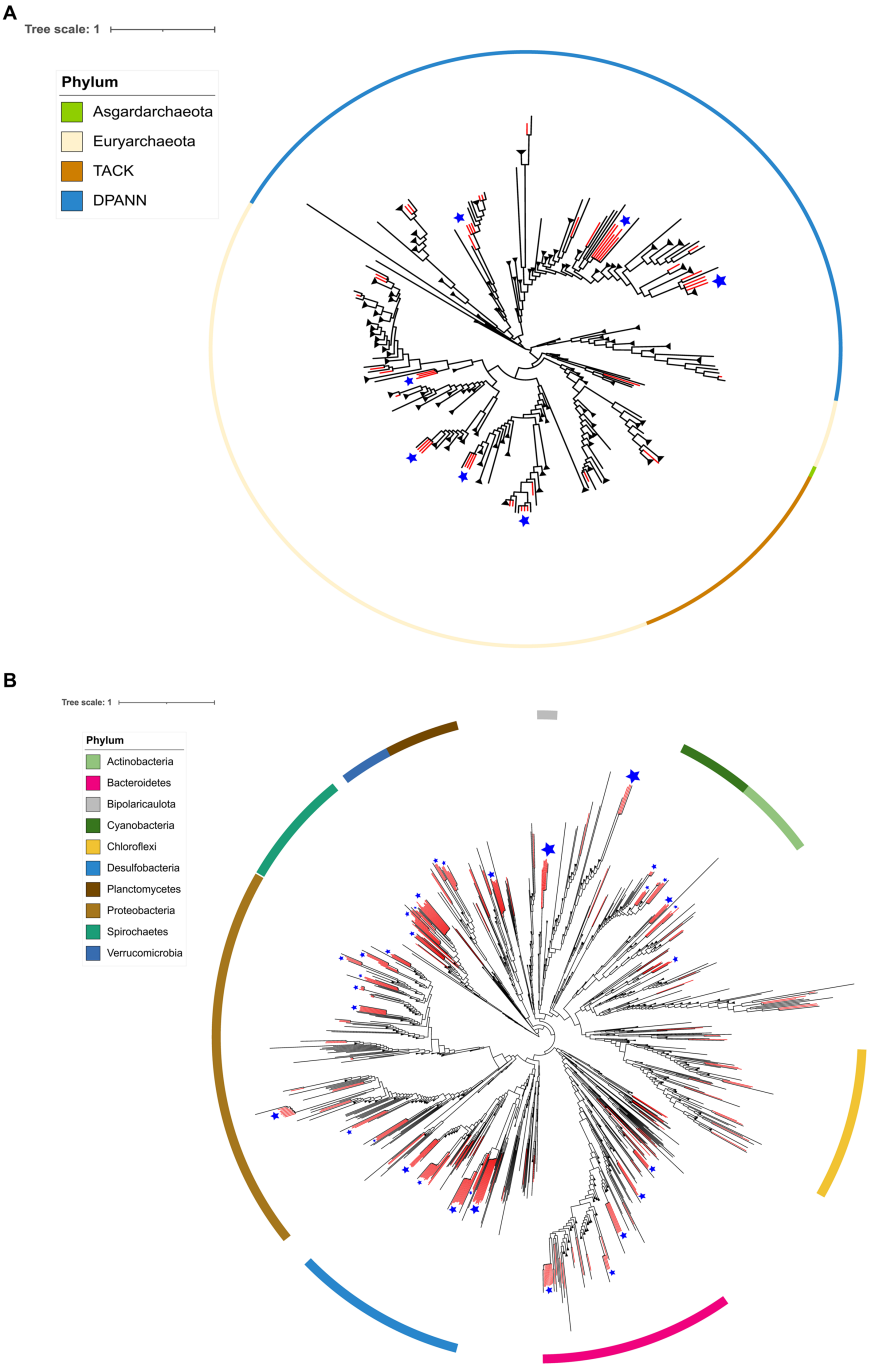
Phylogenomic placement of MAGs from the AD on the Archaea **(A)** and Bacteria **(B)** domain tree. The phylogenomic tree was reconstructed using GTDB-tk. The branches in red show the retrieved AD lineages.

The 48 MAGs assigned to the Archaea domain were taxonomically divided into eleven classes, while the 277 MAGs from the Bacteria domain belong to 47 different taxonomic classes. Figure 4A depicts the Archaea phylogeny, illustrating the distribution of AD Archaea MAGs. It shows that they encompass three Archaeal superphyla: DPANN, TACK, and Euryarchaeota, distributed across 11 different taxonomic classes Archaeoglobi (3), Bathyarchaeia (2), Hadarchaeia (1), Halobacteria (9), Methanosarcinia (1), Micrarchaeia (1), Nanoarchaeia (14), Nanosalinia (2), Aenigmatarchaeia (6), and Thermoplasmata (9).

In the case of Bacteria, Figure 4B depicts the distribution of MAGs, being the most abundant phyla: Proteobacteria (55), Desulfobacteria (53), Spirochaetes (32), Bacteroidetes (28), Cyanobacteria (13), Planctomycetes (12), Verrucomicrobia (11). Other less common phyla are not shown in Figure 4B, including Fibrobacteria, Patescibacteria, Hydrogenedentes, Eremiobacterota, Goldbacteria, Marinimicrobia, Myxococcota, Omnitrophota, Synergistetes, Thermotogota, Acidobacteria, Armatimonadota, Campylobacterota, Gemmatimonadota and Sumerlaeota.

### 3.4 Undestending the functional landscape of MAGs from AD site

**Figure 5:**
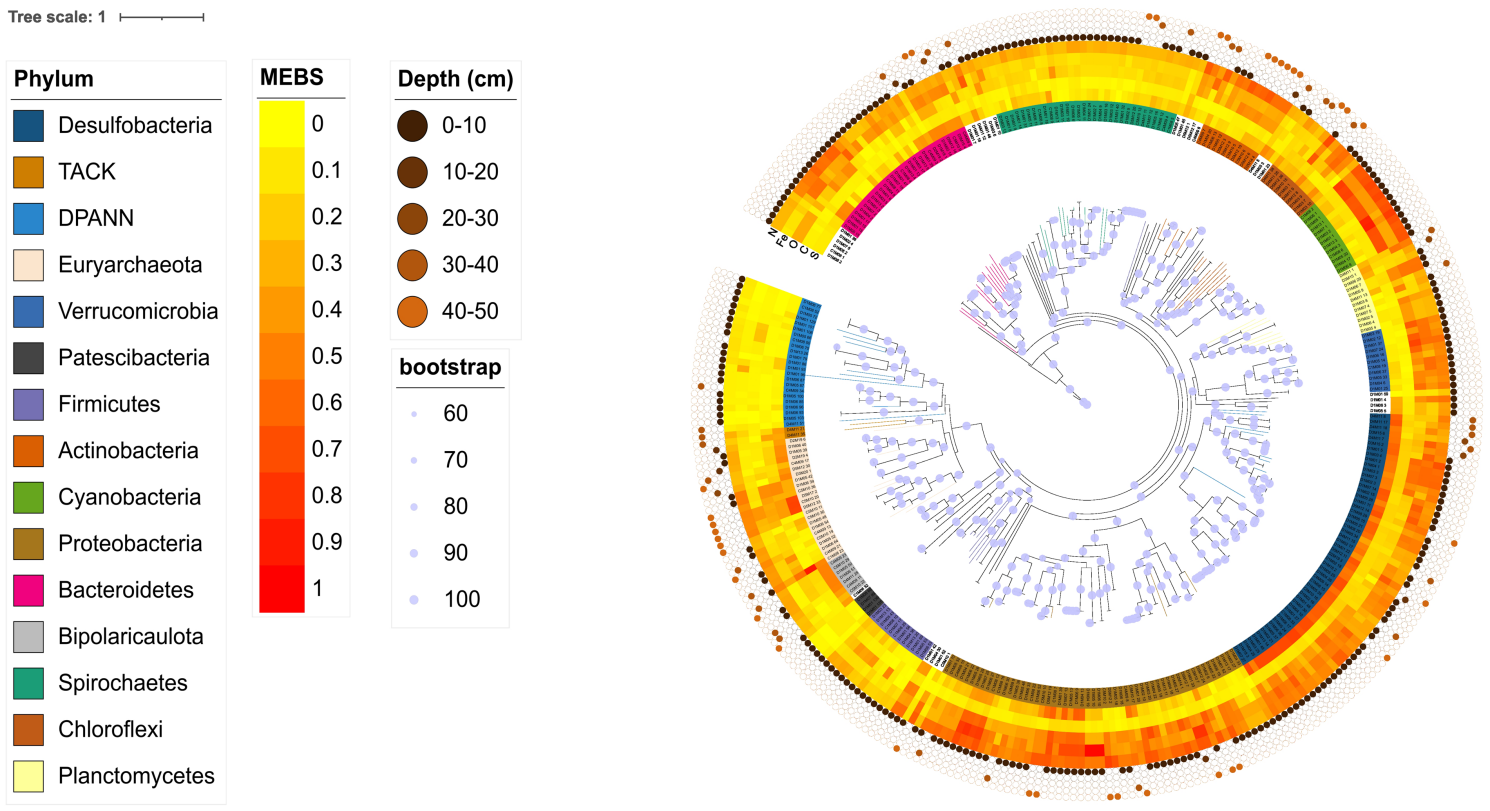
Phylogenomic tree including both Archaeal and Bacterial domains of the unique MAGs from the AD site in CCB. Amino acid substitutions were modeled using the WAG + F + I + R10 model, chosen according to BIC. The concentric circles with different dots show the sample origin, from surface to 40–50 cm. Functional annotation was performed with MEBS software, which uses the relative entropy measure H′(i) to detect enriched protein domains. The obtained H′ values (in bits) capture the extent to which a given Pfam domain informs the metabolism of interest. In this case, domains with H′ values close to or greater than 1 correspond to the most informative Pfam domains, while low H′ values (close to 0) indicate non-informative ones.

The functional annotation of MAGs (Figure 5) shows different metabolic signatures in different microbial phyla. For example, members of the TACK and Euryarchaeota superphyla show an enrichment of genes related to the carbon cycle. Similarly, the desulfobacteria group shows an overrepresentation of genes related to the sulfur cycle, which could be due to their specialization in sulfur-based metabolism. Furthermore, cyanobacteria and proteobacteria show an overrepresentation of genes related to the nitrogen and oxygen cycles, suggesting that these genera are involved in these biogeochemical processes. Finally, certain taxa of the phyla Desulfobacteria, Proteobacteria, Bacteroidetes, Cyanobacteria and Verrucomicrobia have been linked to the iron cycle. Overall, this suggests that microorganisms in the CCB exhibit specific metabolic signatures and specializations.

**Figure 6:**
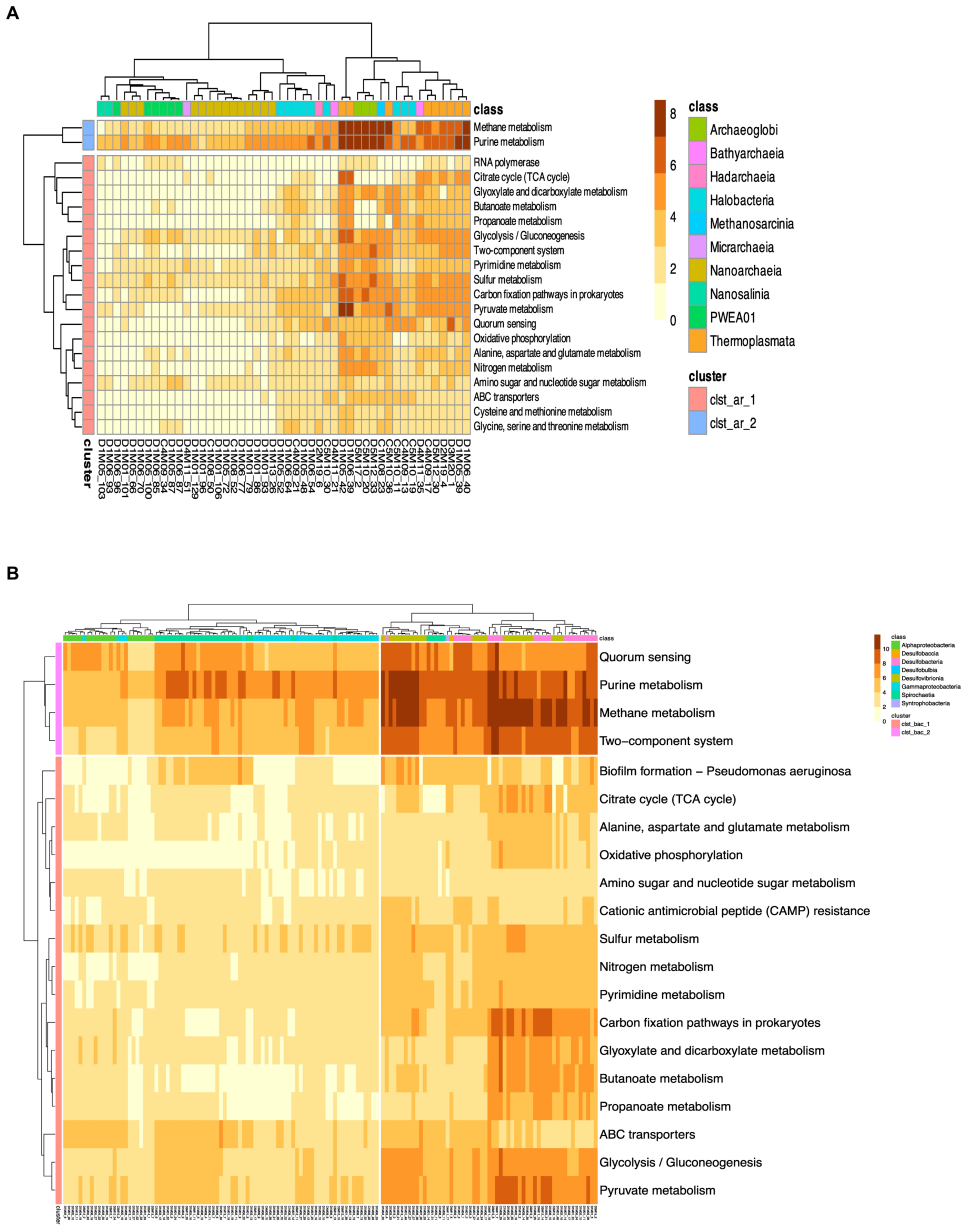
Heat map of the proportion of predicted KEGG orthologs (KO). Panel **(A)** shows the functional annotation for Archeal MAG, while panel **(B)** shows the functional annotation for the most abundant MAGs of the Bacteria domain. The color bar represents the relative abundance of KEGG orthologs (KOs) within each genome, measured as a percentage of the total number of coding sequences. The scale ranges from 0 to 10%, indicating the proportion of KOs relative to the genome’s coding sequences.

We then showed the metabolic landscape of the MAGs found belonging to the above taxa, which are shown in more detail in the heatmap (Figure 6). First, the TACK and Euryarchaeota superphyla shows enriched metabolic pathways such as methane metabolism, purine metabolism, pyruvate metabolism, and sulfur metabolism, that shed light on the metabolic diversity within the archaeal community. Two clusters are formed in Figure 6, with cluster 1 (clst_arc_1) showing the greatest functional diversity. Notable enrichments include amino acid metabolism (alanine, aspartate, cysteine, glycine, glutamate, methionine, serine, threonine), pyrimidine, butanoate, propanoate, pyruvate metabolism and amino sugars. In addition, the prevalence of metabolic pathways such as glycolysis/gluconeogenesis, citrate cycle (TCA cycle) and oxidative phosphorylation emphasizes the metabolic versatility of these archaeal populations in energy production and carbon flux, oxidative phosphorylation, sulfur and nitrogen metabolism as well as in general functions such as genetic information processing (RNA replication), ABC transporters, quorum sensing and two-component systems. While methane metabolism and purine metabolism are more strongly represented in cluster 2 (clst_arc_2). These enrichments are particularly notable in classes such as Thermoplasmata, Archaeglobi, Halobacteria belonging to the phyla of Euryarchaeota found most frequently in AD (17% of abundance), indicating the methane metabolic potential of Euryarcheota within the microbial community.

Cluster 1 (clst_bac_1) of the bacterial MAGs shown in Figure 6B also shows enrichment in multiple metabolic pathways, including amino acid metabolism and carbon fixation, emphasizing the metabolic versatility of bacterial taxa. The identification of metabolic pathways such as glycolysis/gluconeogenesis and the citrate cycle (TCA cycle) indicates active energy metabolism in these bacterial populations. In addition, the presence of genes related to sulfur and nitrogen metabolism highlights their potential contribution to biogeochemical cycling. Overall, these results contribute to our understanding of the metabolic potential and ecological functions of MAGs in the ecosystem. In cluster 2 (clst_bac_2), the metabolic functions with high abundance correspond to purine and methane metabolism, quorum sensing and two-component systems. Since MAGs of the Desulfobacteria phylum account for about 16% (53 of the total 325) of the MAGs reported in this study.

We also performed a Principal Component Analysis (PCA). The PCA revealed clustering patterns like those observed in the heatmaps, (Supplementary Figure S3), where no discernible differences were observed.

The PCA analysis revealed that Dimension 1, explaining 64.9% of the total variance in the data, was dominated by a suite of key metabolic pathways and biological processes. Notable among these pathways were “Methane metabolism,” “Pyruvate metabolism,” “Glycolysis / Gluconeogenesis,” and “Sulfur metabolism,” alongside other pivotal metabolic pathways for microbial life such as “Citrate cycle (TCA cycle),” “Propanoate metabolism,” “Purine metabolism,” and “Pyrimidine metabolism.” Dimension 2, explaining 16.3% of the total variance, exhibited a preponderance of functions related to amino acid metabolism, protein biosynthesis, as well as substrate transport and antimicrobial resistance.

Furthermore, the PCA analysis did not reveal a pronounced segregation between archaeal and bacterial MAGs in terms of their metabolic potential. This suggests that functionally, archaeal and bacterial communities in the dataset may exhibit overlapping metabolic traits or that discrepancies are more nuanced than anticipated. This absence of distinct clustering could stem from the intricate nature of microbial interactions within AD or from the inherent functional heterogeneity of microbial communities existing within this ecosystem.

With the aim of analyzing specific characteristics that highlight diversity in functions, in the microbial community, we chose two MAGs that are among the most abundant phyla in the AD. The MAG C1M08_23 *Methanohalobium* genus (Archaea); it is relevant to mention that in the NCBI database, only one complete genome of this genus is available (*Methanohalobium evestigatum* z-7303) and the MAG D4M11_7 Desulfatiglandaceae (Bacteria) identified only at the family level. For both cases, we generated a metabolic map presented, where observed their functions.

**Figure 7:**
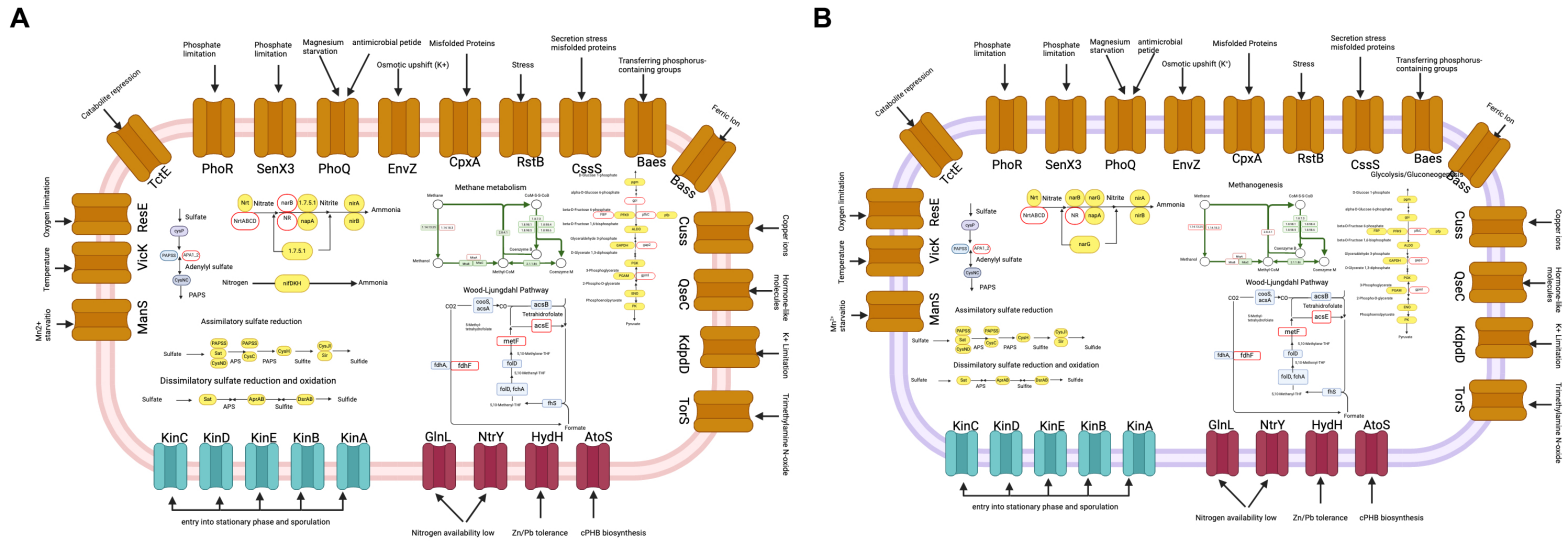
Metabolic map of MAG C1M08_23 of *Methanohalobium* **(A)** and MAG D4M11_7 (family Desulfatiglandaceae) **(B)**. Some metabolic pathways of carbon and nitrogen fixation, and sulfur metabolism are depicted. The red outline ovals denote genes that are absent in the metabolic pathway. The plots were done using biorender.

In Figure 7A, we show metabolic map of MAG C1M08_23, which identifies various two-component systems such as the OmpR and NtrC families associated with sporulation that play a central role in monitoring external conditions and facilitating adaptation to changing environments through two-component signal transduction mechanisms. In addition, genes related to sulfur metabolism involving assimilatory sulfate reduction, dissimilatory sulfate reduction, and oxidation pathways are particularly noteworthy. In particular, the presence of sulfite reductase (ferredoxin), an iron protein critical for sulfate assimilation and the production of cysteine and methionine, emphasizes the metabolic versatility of C1M08_23 MAG. Genes associated with the conversion of extracellular nitrate to ammonium are also observed, as well as two carbon fixation pathways, the Wood-Ljungdahl pathway. Finally, we identified genes related with the methanogenesis pathway like encoding methyl-coenzyme M reductase.

In contrast, the metabolic map of MAG D4M11_7 lacks the gene encoding methyl-coenzyme M reductase, the enzyme that is crucial for the final step of methanogenesis. However, we identified genes encoding heterodisulfide reductase, which is crucial for the energy metabolism of methanogenic archaea. In addition, 7 other genes related to methanogenesis were identified in MAG D4M11_7, including those encoding the tetrahydromethanopterin S-methyltransferase and the MtaC subunit, which is part of a three-enzyme system responsible for catalyzing the formation of methyl coenzyme M. These results showed the metabolic diversity within the microbial community, emphasizing specific metabolic signatures and functional differences between MAGs in AD.

### 3.5 Monophyletic MAG groups suggest endemicity to CCB

Some MAGs within both the Archaea and Bacteria domains exhibit monophyletic clustering patterns at the AD pond, suggesting an endemic nature. We consider a monophyletic cluster, wherein three or more lineages cluster together in the phylogenetic trees generated by GTDB-tk (refer to Figures 4A,B for Archaea and Bacteria domains, respectively). These clusters are denoted by blue star-shaped dots on each phylogenetic tree. Notably, MAGs from various Archaeal phyla such as Euriarchaeota, Candidate Aenigmarchaeota, and Candidate Nanoarchaeota display this clustering pattern (see Figure 4A). Similarly, within the Bacteria domain, phyla such as Actinobacteria, Cyanobacteria, Bipolaricaulota, Spirochaetes, Verrucomicrobiota, Planctomycetes, Bacteroidetes, Desulfobacteria, and Proteobacteria exhibit monophyletic clustering (refer to Figure 4B).

The preceding findings prompted us to conduct separate phylogenetic analyses for three major taxonomic groups, as outlined in the Materials and Methods section: the Candidate phylum Bipolaricaulota, the superphylum PVC (comprising Planctomycetes, Verrucomicrobia, and Chlamydiae), and Cyanobacteria.

**Figure 8:**
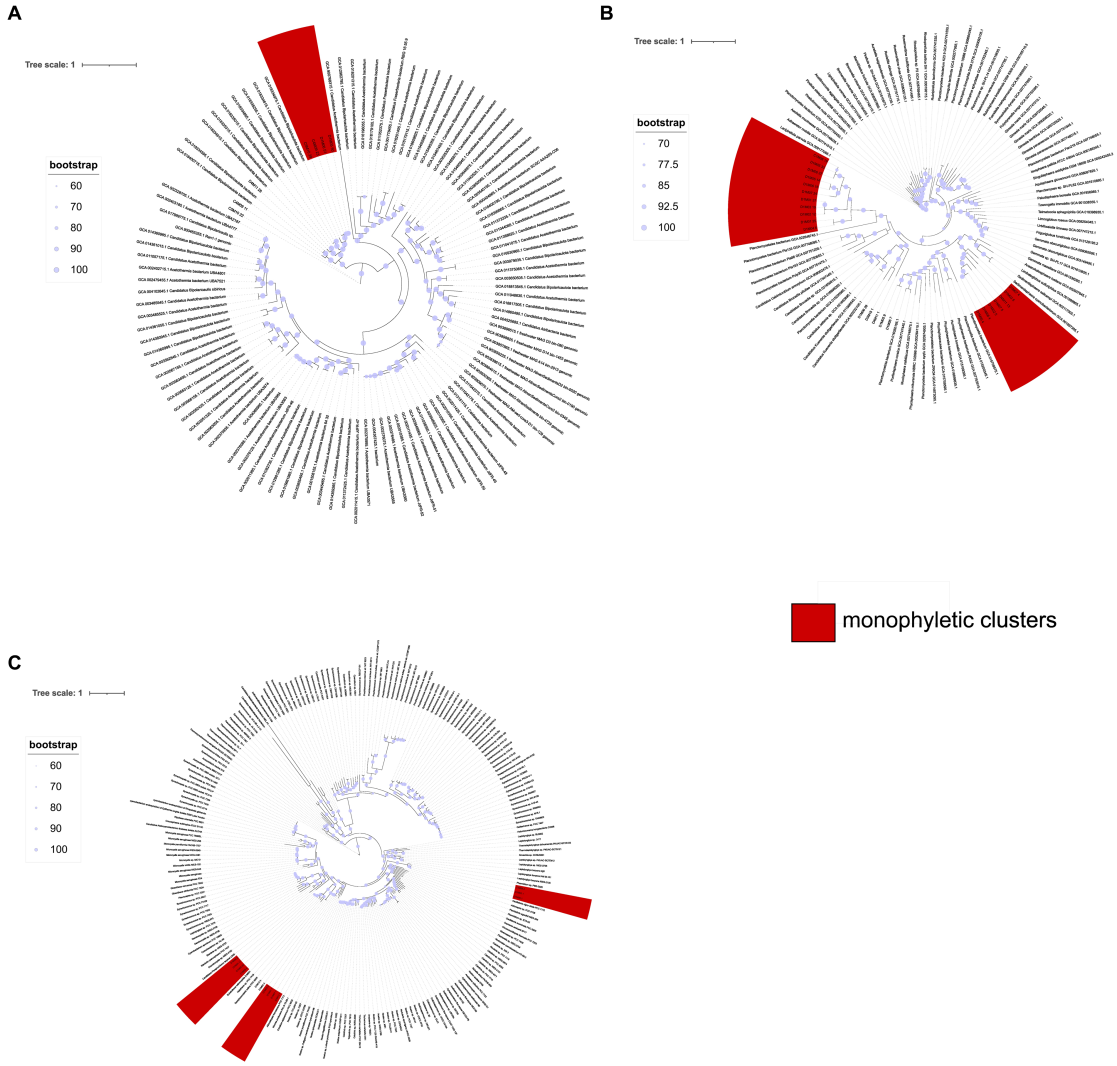
Phylogenetic trees illustrating the evolutionary relationships of the Candidate Bipolaricaulota **(A)**, PVC **(B)**, and Cyanobacteria **(C)** groups, conducted to verify monophyletic groupings. Amino acid substitutions were modeled using the LG + F + R9 model for Candidate Bipolaricaulota, LG + F + R7 for PVC, and LG + F + R5 for Cyanobacteria. The leaves highlighted in red represent individuals from MAGs obtained from the AD site, showcasing a monophyletic grouping.

In the case of the Candidate phylum Bipolaricaulota (Figure 8A), there is a monophyletic group of 4 MAGs (out of a total of 7 MAGs). Likewise, in the analysis of the MAGs of the PVC superphylum (Figure 8B), there are two monophyletic groups, the first one including 7 MAGs, from the Phycisphaerae class, while the other one comprises 11 MAGs of the phylum Verrucomicrobia, order Opituales. The analysis of the cyanobacterial MAGs (Figure 8C) resulted in three monophyletic groups: one including three MAGs from the genus *Sodalinema*; another group of four MAGs from the genus *Coleofasciculus*, and one group of four MAGs from the genus *Halothece*.

The observation of monophyletic groups in the phylogenetic trees of the three groups analyzed could denote organisms more closely related to each other than to external entities, which share unique characteristics absent in distant ancestors. Monophyletic clusters could suggest a shared ancestry among the included species. If these clusters are found in specific geographic regions, it suggests that the common ancestor of those species was also present in this area. Over time, if these species have evolved in isolation, either due to geographical barriers or ecological niches, they may give rise to unique and endemic lineages within that region, as already demonstrated by Alcaraz et al., (2008), Souza et al. (2018).

Finally, a metagenomic recruitment analysis was conducted, and due to limitations in displaying all graphs, Supplementary Figure S2 shows the results of the comparison of the reference genome of *Halothece* sp. PCC 7418 (formerly *Aphanothece halophytica*). This marine cyanobacterium is indigenous to extreme saline environments and can thrive in salt concentrations of up to 3.0 M NaCl (Soontharapirakkul et al., 2011). Notably, the analysis revealed multiple regions of low coverage across the genome, as depicted in the top right panel of Supplementary Figure S2. Additionally, the base pair Pileup plot did not show a maximum peak at 100%, but rather at 88.2%. These findings suggest that while some MAGs are classified as *Halothece*, forming a monophyletic group as observed in the phylogenetic analysis, MAGs from the AD site exhibit greater phylogenetic similarity to each other than to microorganisms from other sites, as evidenced by the comparison with *Halothece* sp. PCC 7418.

## 4 Discussion

### 4.1 Taxonomic composition: bacteria prevail over archaea in the AD site

The taxonomic analysis of the Archean Domes (AD) site in the Cuatro Ciénegas Basin (CCB) revealed a diverse population of archaea with a relative abundance of 14.45%, especially the phylum Euryarchaeota. This contrasts with other CCB locations, where Archaea typically represent only about 2% of the microbial community (Souza et al., 2008; Bonilla-Rosso et al., 2012). This finding highlights the uniqueness of the Archean Domes site, as previously suggested by (Espinosa-Asuar et al., 2022; Medina-Chávez et al., 2023; Madrigal-Trejo et al., 2023). Euryarchaeota.

An alternative to the diversity of archaea could be explained by possible movements of the deep aquifers that brought microorganisms from the deep biosphere to the surface. This could have influenced the microbiological diversity at the AD site and in other ponds within the CCB. The studies by Madrigal-Trejo et al. (2023), Cisneros-Martínez et al. (2023) and Wolaver et al. (2012) have also observed the possible connection and influence of the movement of deep aquifers.

The diversity of archaea at the AD site can be attributed to the salinity conditions present in the environment. The Euryarchaeota is the most predominant, as observed in other places with high salinity (Fernández et al., 2014; Wang et al., 2022). However, bacteria are generally more diverse than archaea. Taxa such as Proteobacteria, Firmicutes, Bacteroidetes, Actinobacteria, and Cyanobacteria are among the most common.

According to Nonpareil curves, our metagenomes have an average coverage of 60 to 70%. However, some samples have even lower coverage, reaching 20% in some cases. This implies that there is still a significant proportion of diversity to explore in our samples. For example, has been reported that the soil samples required greater sequencing effort to achieve almost complete coverage, suggesting their complexity (Rodriguez-r and Konstantinidis, 2013). Nonpareil curves are useful in revealing distinctive characteristics of samples, such as the skewed distribution of species abundance, and tell us when a sufficient proportion of the diversity present in the sample has been sequenced. In our case, some samples barely reach 20% coverage, suggesting much to discover.

### 4.2 Taxonomic novelty of AD site

This is the first time the metagenome-assembled (MAG) genomes were used to expand our understanding of the CCB prokaryotic tree of life, unlike previously published studies that used 16S rRNA tags or metagenome assemblies. Our analysis revealed the presence of several predominant phyla, including Proteobacteria, Cyanobacteria, Firmicutes, Bacteroidetes, Actinobacteria, Spirochaetes, Chloroflexi, and Euryarchaeota, which is consistent with previous studies of diversity at the AD site (Espinosa-Asuar et al., 2022; Madrigal-Trejo et al., 2023).

As mentioned in the preceding paragraphs, the AD site harbors microorganisms that render this environment unique. As an example, we obtained the MAG D1M01_16 classified as Marinisomatota phylum, which is a recently proposed bacterial candidate phylum formerly known as SAR406, MGA, or Marine Group A. These bacteria are predominantly found at great depths, such as the Challenger Deep, the Mariana Trench, and the Puerto Rico Trench (Tarn et al., 2016). This phylum exhibits low representation in shallow pelagic samples and high abundance in deep samples. Although these bacteria are often associated with low levels of dissolved oxygen environments, little is known about their ecology and metabolic functions. Marinisomatota is part of the FCB group, alongside other related bacterial phyla.

This MAG, along a wealth of data obtained since 2000 (Souza et al., 2006, 2012, 2018; Alcaraz et al., 2008) supports marine ancestry of CCB despite geological indications that marine waters left the valley with the uplift of the Mexican Sierra Madre Oriental and the closure of the Wester Sea Way 35 MYA. Thus, the CCB has two of the ingredients for hyper-diverse microbial endemism: isolation and long-term continuity.

Moreover, these MAGs have enabled us to identify 12 high-quality genomes, as per the MIMAG criteria. Among these, only two MAGs, D1M03_19 and D1M06_10, were classified at the genus level: *Puniceicoccus* and *Wenzhouxiangella*, respectively. Moreover, within this set of genomes, the presence of three high-quality genomes from the Archaea domain is noteworthy: D4M11_35 (class Bathyarchaeia), D1M06_39 (order Methanomassiliicoccales), and D4M11_51 (family Bilamarchaeaceae). Given the limited number of reported Archaea genomes in the NCBI database (i.e., only 581 genomes reported as completely sequenced, as of the manuscript writing date), this study makes a substantial contribution to enhancing the taxonomic sampling within the Archaea domain.

Moreover, most of the MAGs found in this study represent —at least at the species level—previously unknown taxa. This is not surprising, considering that many of the phyla in which our MAGs are classified have only recently been described. For instance, the Candidate Aenigmarchaeota is an archaeal cluster first identified in 2013 as part of a study of “microbial dark matter” (Rinke et al., 2013). The same is true for the Candidate Woesearchaeota phylum and which was also recently described (Castelle et al., 2015).

The diversity within CCB AD site is notable, demonstrated by the distribution of its MAGs across nearly the entire prokaryotic tree of life, spanning both Bacteria and Archaea (refere to Figures 4–6). In comparison to other hypersaline sites, only Shark Bay (blue hole mats) (Kindler et al., 2021) and Lake Hillier (Sierra et al., 2022) have reported members within the Asgard, TACK, DPANN, and Euryarchaeota superphyla coexisting within the same microbial mat environment. This highlights the unique nature at the AD site and its potential as a source to study microorganisms’ evolution and adaptation in extreme environments. Euryarchaeota, Asgard, and DPANN were also reported in Guerrero Negro (García-Maldonado et al., 2022). Euryarchaeota, TACK, and DPANN presence has been reported in High-Bourne Cay (Khodadad and Foster, 2012). Previous studies have revealed varying compositions of prokaryotic communities across different hypersaline environments. For instance, in Lake Magadi (Kambura et al., 2016) and Hamelin Pool (Ruvindy et al., 2015), reports have primarily focused on Euryarchaeota and TACK members. In contrast, Cape Recife (Waterworth et al., 2020) exhibits a broader diversity, including Asgard and DPANN members. However, some analyses have primarily highlighted the dominant relative abundance of specific taxa within single phyla. For instance, in the hypersaline pool Lake Tyrrell (Andrade et al., 2015), *Haloquadratum* species and unculturable members of Halobacteriaceae are predominant. Similarly, in the Dead Sea (Jacob et al., 2017), Euryarchaeota and Nanohaloarchaeota dominate. At the Hammam Essalihine site (Adjeroud et al., 2020), the representation of Archaea is relatively weak, primarily comprising members of Parvarchaeota and Crenarchaeota. Meanwhile, in the Salar de Atacama sites (Laguna Brava and Tebenquiche), (Kurth et al., 2021), Lago Diamante (Rascovan et al., 2015), Socompa (Kurth et al., 2017), and Rottnest Island (Mendes Monteiro et al., 2020), only representatives of Euryarchaeota have been reported.

### 4.3 Understanding the functional landscape of MAGs from AD site

The functional annotation of MAGs obtained from AD suggests that certain members of the Archaea play a fundamental role in the carbon cycle. For example, MAG C1M08_23 has the highest level of genes associated with the carbon cycle. Its taxonomic classification according to GTDB-tk corresponds to the Euryarchaeota Phylum, genus *Methanohalobium*. It is worth mentioning that this genus contains only one species described so far: *M. evestigatum* (Zhilina and Merkel, 2019), which is halophile and extremely thermophilic, and lives in the hypersaline lagoons of the Arabat spit (East Crimea). It has been reported that this Archaea lives exclusively on the production of methane, either by reducing carbon dioxide with hydrogen or by using methyl compounds as substrates.

In the sulfur cycle context, it is evident that Bacteria within the Desulfobacteria phylum play a central role. Out of 325 MAGs, 53 belong to this phylum, distributed across different taxonomic orders: 26 Desulfovibrionales, 20 Desulfobacterales, 4 Desulfatiglandales, 1 Desulfobulbales, 1 Desulfobaccales and a MAG from the class Syntrophobacteria. It has been reported that almost all bacteria from these orders are sulfate-reducing microorganisms, that is, they can perform anaerobic respiration utilizing sulfate as terminal electron acceptor, reducing it to hydrogen sulfide (Muyzer and Stams, 2008; Ward et al., 2021). It has also been suggested that they may have contributed to the sulfur cycle shortly after the origin of life on Earth, making them potential ancestors of many microorganisms in a geological context (Wasmund et al., 2017).

Regarding the oxygen and nitrogen cycles, cyanobacteria are expected to have an overrepresentation of genes associated with these cycles. This expectation stems from the fact that cyanobacteria are primary producers (Chen et al., 2022). This primary productivity usually occurs through photosynthesis, which uses light as an energy source (Hamilton et al., 2015). However, primary productivity can also occur through chemoautotrophy, which uses the oxidation or reduction of inorganic chemical compounds as an energy source (Stal, 2012). For instance, out of the 13 cyanobacterial MAGs reported in this study, four were classified within the genus *Coleofasciculus*. Microorganisms of this genus have been reported as one of the most abundant in the microbial mats of the hypersaline lagoon system of Araruama in Brazil (HLSA) (Walter et al., 2021). This suggests that the high abundance of microorganisms of this genus is likely due to their tolerance to high saline levels and their metabolic flexibility (i.e., ability to perform both photosynthesis and anoxic fermentation) (Burow et al., 2012; Walter et al., 2021).

On the other hand, MAGs D1M13_3 and D1M04_3 classified within the Spirulinaceae family have the highest MEBS index values associated with oxygen cycle genes. These MAGs are phylogenetically close to the cyanobacterial strain ESCF-1, which has been shown to be an important diazotroph in the intertidal microbial mat system in Elkhorn Slough (Everroad et al., 2016), and it has been shown to produce a considerable external carbon pool in the form of EPS (Extracellular polymeric substances). This EPS are managed by an active exoproteome and provides a source of organic carbon for cyanobacteria and other community members (Stuart et al., 2015).

Genes related to the iron cycle are also overrepresented in members of Proteobacteria. For example, in MAGs D1M05_10, D1M04_19, and D1M06_10, all of which belong to the genus *Wenzhouxiangella*, these genes are well represented. Some isolates of this genus were obtained from environments with physicochemical conditions like those described in AD, including alkaline pH and high salt concentration. An example of such isolates is the *Wenzhouxiangella* strain AB-CW3 (Sorokin et al., 2020), which was obtained from a system of hypersaline alkaline soda lakes in the Kulunda steppe. In this strain, the presence of mtrAB-like genes was reported, which are part of an electron transport system known for iron-reducing bacteria. In this context, these genes may play a role in iron uptake (Sorokin et al., 2020).

It is evident that the Archaea belonging to the DPANN superphylum, due to their reduced genomes, do not have an overrepresentation of genes related to any of the C, N, O, S, or Fe cycles. These organisms are characterized by limited metabolic capabilities, with both catabolic and anabolic capacities being significantly limited (Dombrowski et al., 2019). This suggests that at least some members of this superphylum may function as obligate symbionts.

The PCA analysis conducted in this study provides crucial insights into the functional diversity of microbial communities within the AD. Dimension 1, explaining 64.9% of the variance, highlights the significant contribution of metabolic pathways like “Pyruvate metabolism,” “Glycolysis/Gluconeogenesis,” and “Methane metabolism” to ecosystem functioning. This underscores the metabolic versatility of microbial communitie inhabiting the AD and their pivotal role in driving biogeochemical processes.

Of particular interest is the high loading of “Sulfur metabolism” on Dimension 1, emphasizing the importance of sulfur cycling mediated by microbial communitie in the AD. Dimension 2, explaining 16.3% of the variance, reveals distinct patterns of functional variation, with pathways related to amino acid metabolism and antimicrobial resistance playing key roles in functional differentiation among microbial communities within the AD (Band and Weiss, 2014).

The significant loading of “Methane metabolism” underscores the importance of methane as a primary carbon and energy source in AD site. Methanogenic archaea and methane-oxidizing bacteria are central to methane cycling, influencing ecosystem dynamics (Kharitonov et al., 2021).

The absence of clear separation between archaeal and bacterial MAGs in terms of their metabolic potential raises intriguing questions about functional redundancy and ecological roles within the AD. This suggests potential functional redundancy within microbial communitie or niche partitioning, warranting further investigation.

Regarding the functional annotation of MAG C1M08_23 (*Methanohalobium*), it is important to note that, as mentioned in the results, in the NCBI database, there is only one fully sequenced genome of this genus (*Methanohalobium evestigatum* Z-7303) and another at the scaffold level (with assembly identifier GCA_018609725.1). Thus, this work contributes to a better understanding of the metabolism and ecology of the archaeal genus *Methanohalobium*, which has been reported to be strictly anaerobic and exclusively sustains itself through methane production via the reduction of carbon dioxide with hydrogen or by utilizing methyl compounds as substrates. These species are only moderately halophilic but extremely thermophilic.

Based on the results obtained in this study, we can suggest that it is a potentially methanogenic archaea capable of fixing carbon through the Wood-Ljundahl pathway and possibly able to fix nitrogen. This capability, on the other hand, provides a broader perspective in the field of nitrogen fixation. Biochemical and genetic studies demonstrate that nitrogen fixation in Archaea is evolutionarily related to nitrogen fixation in Bacteria and operates through the same fundamental mechanism (Leigh, 2000; Gaby and Buckley, 2014). At least three nif genes present in Bacteria (nif H, D, and K) are also found in MAG C1M08_23, suggesting that it may be a diazotrophic methanogenic archaea.

Furthermore, this genome suggests the capability for dissimilatory sulfur reduction, as well as dissimilatory sulfur reduction and oxidation. Finally, as shown in Figure 7B, multiple two-component systems were found, which could assist this microorganism in thriving in an extreme environment such as the AD pond. For instance, this MAG encodes for PhoR and SenX3 (James et al., 2012), reported to be involved in the regulation of gene expression under phosphorus-limiting conditions. *Methanohalobium* also encodes for multiple histidine kinases, such as KinABCDE, which regulate entry into the stationary phase and sporulation, possibly homologous to genes already reported in *Bacillus subtilis* (Tojo et al., 2013). Additionally, this MAG encodes for NtrY and GlnL, two two-component systems of the NtrC family related to conditions of low nitrogen availability. Regarding the metabolic description of MAG D4M11_7 (Desulfatiglandaceae family), it is suggested that this microorganism has the capability to perform assimilatory sulfur reduction, as well as dissimilatory sulfur reduction and oxidation. Additionally, it incompletely harbors genes associated with the carbon fixation cycle through the Wood-Ljungdahl pathway. However, it is not possible to conclusively state whether this organism is fully capable of executing this pathway, if the genes are present as vestiges, or if the missing genes necessary to complete the pathway are absent due to assembly challenges (87.49% according to CheckM, refer to this value and other values related to MAG quality in Supplementary Table S4) or a true lack of the genes in the MAG.

Likewise, it is important to note that this MAG encodes for some genes related to methanogenesis, such as the heterodisulfide reductase (HDR). This enzyme, crucial in the Wolfe cycle of methanogenic archaea that generate methane from CO2 and H2, catalyzes the reduction of heterodisulfide (CoM-S–S-CoB) to coenzyme M (CoM-SH) and coenzyme B (CoB-SH). Additionally, it encodes for the enzyme tetrahydromethanopterin S-methyltransferase, which catalyzes the transfer of methyl groups from methyl-tetrahydromethanopterin to 2-mercaptoethane-sulfonate and has been identified in the methane-synthesizing complex of *Methanobacterium thermoautotrohicum*. However, we have not found evidence that this gene encodes for methyl coenzyme M reductase (MCR), which catalyzes the terminal step in biogenic methane production (Aguinaga Casañas et al., 2015).

According to the functional annotation of this MAG, it is possible that this microorganism could convert extracellular nitrate to ammonium. The involvement of microorganisms from the order Desulfobacterales in the nitrogen cycle has been previously demonstrated, as the nitrate reduction by Desulfobacterales has been observed to efficiently alleviate nitrogen pollution in the subtropical mangrove ecosystem in the Beibu Gulf in China (Nie et al., 2021). Like the MAG C1M08_23 of *Methanohalobium*, MAG D4M11_7 encodes for multiple two-component systems such as PhoR and SenX3, also present in MAG C1M08_23, along with multiple histidine kinases such as KinABCDE that regulate entry into stationary phase and sporulation, as well as NtrY and GlnL, which are two two-component systems of the NtrC family related to conditions of low nitrogen availability.

### 4.4 Monophyletic MAG groups suggest endemicity to CCB

The AD site exhibited the presence of the recently described candidate phylum Bipolaricaulota (Hao et al., 2018), which showed monophyletic clustering. Similarly, our phylogenetic analysis of the cyanobacteria group and PVC also showed the formation of such monophyletic groups. These findings suggest that the groups analyzed, as well as other groups showing similar phylogenetic patterns, likely represent endemic groups. The observed clustering pattern could be related to oligotrophic conditions characterized by limited phosphorus availability reported at the site, as evidenced by a reported C:N:P ratio of 122:42:1 (Espinosa-Asuar et al., 2022; Medina-Chávez et al., 2023; Madrigal-Trejo et al., 2023).

Previous studies have suggested that the low phosphorus (P) and other conditions in CCB have triggered an evolutionary response among its endemic microorganisms (Souza et al., 2018) exemplified by *B. coahuilensis*. Remarkable adaptations to the environment have been observed in *B. coahuilensis*, including its ability to produce sulpholipids instead of phospholipids (Alcaraz et al., 2008). This adaptation is attributed to the absence of genes responsible for synthesizing P-rich teichoic acids and polyanionic teichuronic acids (Souza et al., 2008).

Furthermore, we conducted a recruitment analysis using 17 genomes reported as reference in the NCBI database (refer to Supplementary Table S4 for more information on these genomes). This analysis revealed minimal genome coverage when compared to the reference genomes, suggesting that these MAGs possess unique genomic characteristics not found in previously reported genomes. These findings align with our observations of the phylogenetic similarity of MAGs from the AD site and their distinctiveness from microorganisms found at other locations, such as the comparison with *Halothece* sp. PCC 7418.

## 5 Conclusion

We described here 325 MAGs from the AD site, comprising both Archaea (48) and Bacteria (277), spanning remarkably 40 phyla across both domains. The AD site displays high salinity and fluctuating pH and has been of interest since its discovery in 2016 because of its unique physicochemical conditions that support the growth of extremophile organisms.

Our study provides information on the remarkable diversity and unique characteristics of microorganisms at AD, and the MAGs reported here enhanced our understanding of the prokaryotic tree of life, revealing a diverse microbial community, which, viewed from a phylogenetic perspective, suggests that the AD site might harbor many endemic lineages. The study highlights the exceptional microbiological diversity in this environment, as none of the MAGs could be classified at the species level, and a significant portion (126 MAGs) could not be classified even at the genus level. These results strongly suggest the presence of previously unknown microbial species and genera at this site. Phylogenetic analysis also reveals monophyletic clustering patterns, which could suggest that microorganisms at the AD site are endemic to CCB.

We consider that the collection of MAGs obtained in this study will serve as a valuable resource for expanding the knowledge of microbial diversity within the tree of life.

## Data availability statement

The data presented in the study are deposited in the NCBI repository, in the bioproject number PRJNA847603.

## Author contributions

UR-C: Conceptualization, Data curation, Formal analysis, Investigation, Methodology, Software, Writing – original draft, Writing – review & editing. HC-S: Investigation, Writing – original draft, Writing – review & editing. DM-T: Writing – review & editing. LE: Conceptualization, Funding acquisition, Project administration, Resources, Validation, Visualization, Writing – original draft, Writing – review & editing. VS: Funding acquisition, Project administration, Resources, Supervision, Validation, Visualization, Writing – original draft, Writing – review & editing.

## Footnotes

^1^ http://langebio.cinvestav.mx/labsergen/

## Supplementary material

SUPPLEMENTARY FIGURE S1 Nonpareil curves of the metagenomes of CCB. The x-axis shows the sequencing effort in bp (base pairs) and the y-axis shows the estimated average coverage, with each metagenome shown in a different color. The circles on the curve represent the average coverage of each sample.

SUPPLEMENTARY FIGURE S2 Recruitment plot displaying D1M06 metagenome reads mapped to a single *Halothece* sp. PCC_7418 reference genome. **(A)** is a 2-D histogram displaying the percent identity of reads to the reference genome on the y-axis and the position in the genome on the x-axis. Cell fill color darkens as more reads fall within the cell **(B)** is a line plot of the average depth of coverage per genome region on the main panel. The dark blue line displays depth of coverage for reads mapping to regions of the reference genome **(A)**, and the light blue line displays depth for reads outside this population. Note the logarithmic scale in the base pair counts axis as well as the highlighted area of lower coverage, representing a reference genomic region not shared by the majority of the metagenomic population. **(C)** is a histogram of depths of coverage across the entire genome **(D)** is a histogram of the number of bases displayed in panel **(A)** (x-axis) which fall into percent identity windows (y-axis), here displayed in log scale.

SUPPLEMENTARY FIGURE S3 A principal component analysis conducted on the functional annotation of MAG (PC1 explaining 64.9% of the variance; PC2 explaining 16.3% of the variance).
