# Supplementary figures and images for: Uncovering novel bacterial and archaeal diversity: genomic insights from metagenome-assembled genomes in Cuatro Cienegas, Coahuila
Source: Front Microbiol. 2024 May 30;15:1369263. doi: 10.3389/fmicb.2024.1369263 (PMC11169877; doi:10.3389/fmicb.2024.1369263)

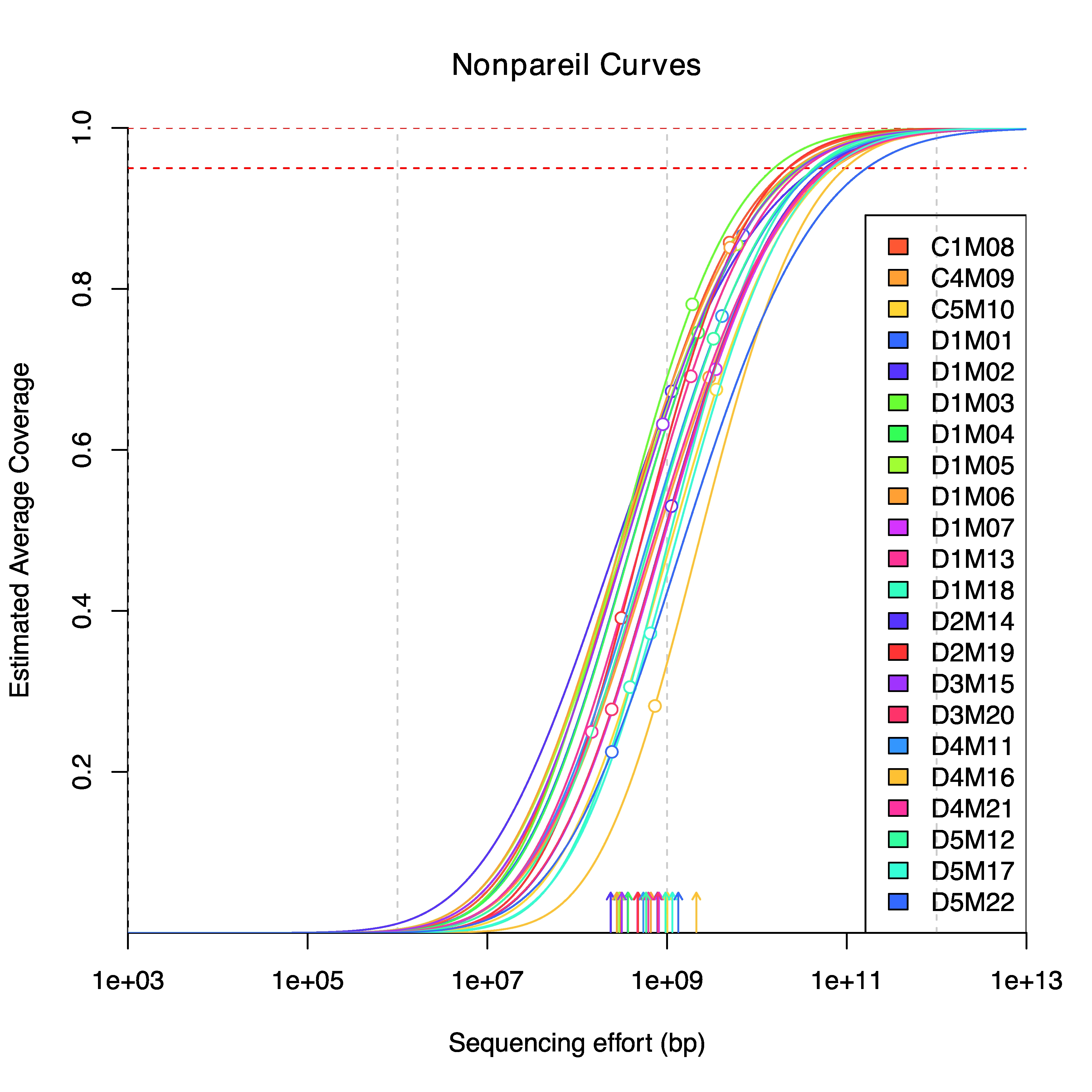

Supplement: SUPPLEMENTARY FIGURE S1 — Nonpareil curves of the metagenomes of CCB. The x-axis shows the sequencing effort in bp (base pairs) and the y-axis shows the estimated average coverage, with each metagenome shown in a different color. The circles on the curve represent the average coverage of each sample. [file Image_1.JPEG]

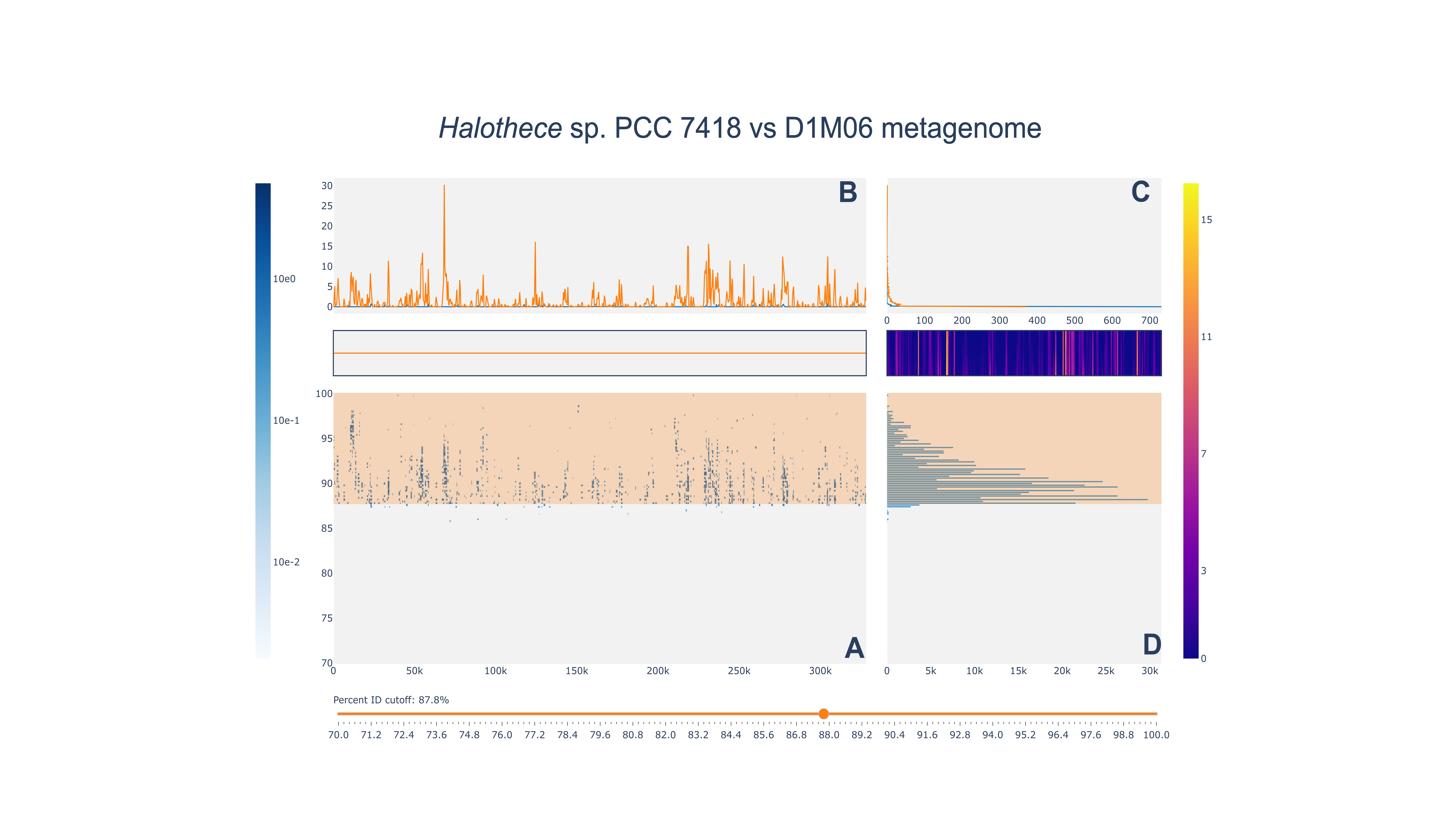

Supplement: SUPPLEMENTARY FIGURE S2 — Recruitment plot displaying D1M06 metagenome reads mapped to a single Halothece sp. PCC_7418 reference genome. (A) is a 2-D histogram displaying the percent identity of reads to the reference genome on the y-axis and the position in the genome on the x-axis. Cell fill color darkens as more reads fall within the cell (B) is a line plot of the average depth of coverage per genome region on the main panel. The dark blue line displays depth of coverage for reads mapping to regions of the reference genome (A), and the light blue line displays depth for reads outside this population. Note the logarithmic scale in the base pair counts axis as well as the highlighted area of lower coverage, representing a reference genomic region not shared by the majority of the metagenomic population. (C) is a histogram of depths of coverage across the entire genome (D) is a histogram of the number of bases displayed in panel (A) (x-axis) which fall into percent identity windows (y-axis), here displayed in log scale. [file Image_2.JPEG]

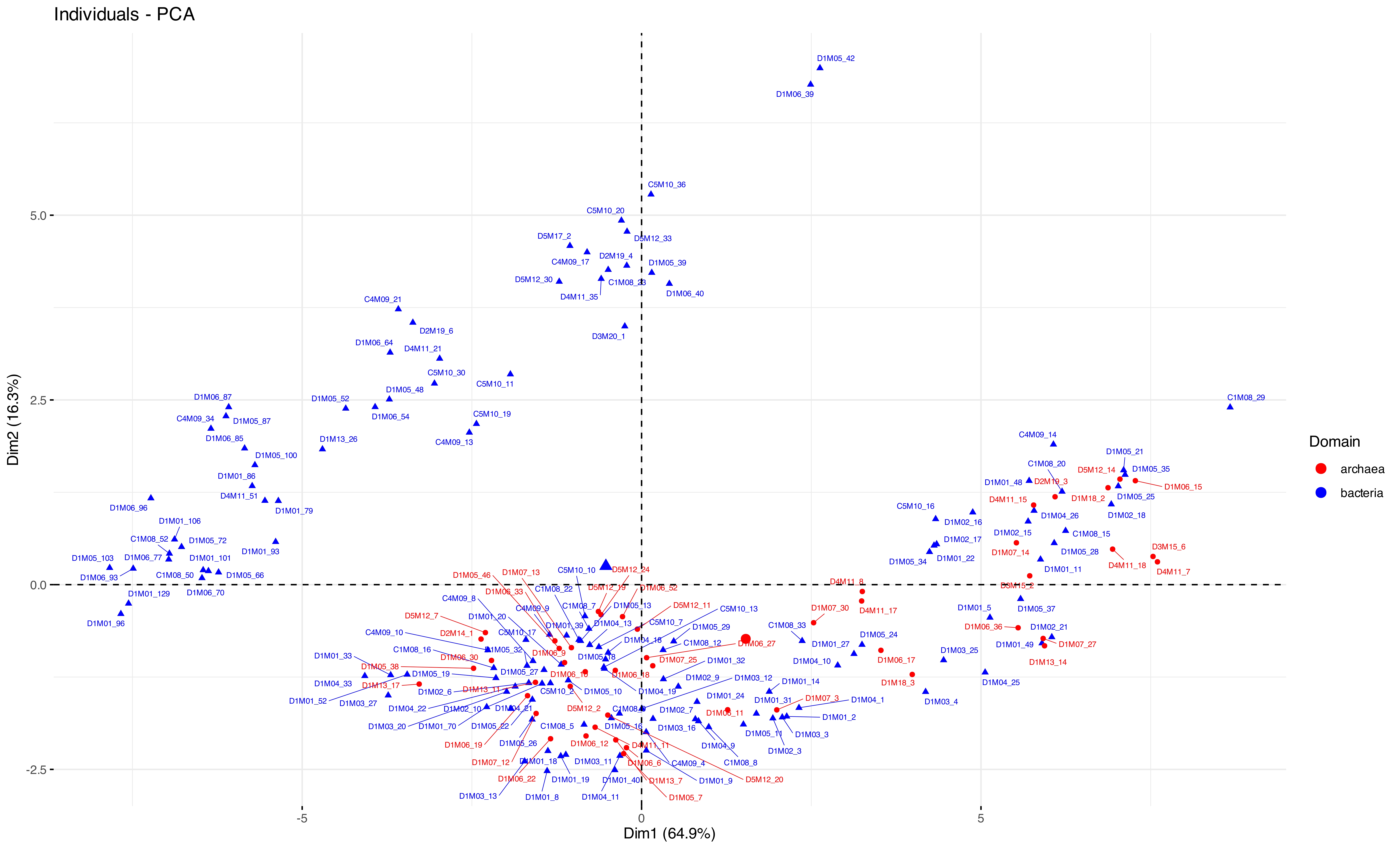

Supplement: SUPPLEMENTARY FIGURE S3 — A principal component analysis conducted on the functional annotation of MAG (PC1 explaining 64.9% of the variance; PC2 explaining 16.3% of the variance). [file Image_3.JPEG]
